# Post‐COVID‐19 vaccine acute encephalitis in an adult patient: A case report and literature review

**DOI:** 10.1002/ccr3.6915

**Published:** 2023-02-08

**Authors:** Maryam Pakfetrat, Leila Malekmakan, Bijan Najafi, Taraneh Zamani, Mina Mashayekh

**Affiliations:** ^1^ Department of Community Medicine, Shiraz Nephro‐Urology Research Center Shiraz University of Medical Sciences Shiraz Iran; ^2^ Internal Medicine Department Larestan University of Medical Sciences Larestan Iran

**Keywords:** COVID‐19 vaccine, encephalitis, hyponatremia, SADH, Sinopharm BIBP

## Abstract

Several vaccines were approved after COVID‐19 pandemic, which have been fast‐tracked for emergency use. The short‐ and long‐term safety profile has been an area of concern. We presented a patient with encephalitis followed by hyponatremia who developed hallucination and seizure 1 day after receiving the second dose of Sinopharm vaccine.

## 1 INTRODUCTION

In December 2019, a cluster of acute respiratory illnesses that led to a pneumonia outbreak occurred in China, which was caused by severe acute respiratory syndrome coronavirus 2 (SARS‐CoV‐2)^1^ Since then, vaccine production has tremendously accelerated, leading to the developing of multiple new and effective vaccines against COVID‐19 in a relatively short period.^2^ However, because vaccines have been fast‐tracked for emergency use, the short‐ and long‐term safety profile has been an area of concern.^3^ Among neurological manifestations related to the COVID‐19 vaccines, some like Bell's palsy, headache, cerebrovascular events, or Guillain–Barré syndrome have been reported more frequently^4^; however, some others such as Vaccine‐induced immune thrombotic thrombocytopenia (VITT)^5^, ^6^ or acute encephalopathy^7^, ^8^, ^9^ considered as rare.

This paper aims to introduce a patient who developed encephalitis after receiving the second dose of the COVID‐19 Sinopharm vaccine and review literature regarding post‐COVID‐19 vaccination encephalitis complications for early diagnosis and treatment.

## 2 CASE PRESENTATION

A 65‐year‐old previously healthy man was admitted to the emergency department for evaluation of hallucination started 1 day before admission. The patient has been relatively well until 1 week prior to admission, developing upper respiratory tract symptoms including rhinorrhea, headache, malaise, and low‐grade fever. The COVID‐19 polymerase chain reaction (PCR) test was negative. After that, the patient received the second dose of Sinopharm vaccine (the first dose was also Sinopharm) 3 days before admission and developed high‐grade fever, hallucination, and disorientation to time, place, and person 24 h later. The timeline for course of events is provided in Table 1. The patient was not oriented to time, place, or person. Vital signs showed blood pressure 120/80 mmHg, pulse rate 80 bpm, and temperature 38°C. Physical examination was remarkable for normal cardiac, respiratory, and abdominal examinations. The neurologic examinations were normal except for disorientation.

**TABLE 1:** Timeline of course of events.

Event	Time
Prodromal symptoms	December 7, 2021
COVID‐19 PCR test	December 7, 2021
Result of COVID‐19 PCR test (negative)	December 12, 2021
Getting the second dose of Sinopharm vaccine	December 11, 2021
Admission	December 14, 2021

**FIGURE 1:**
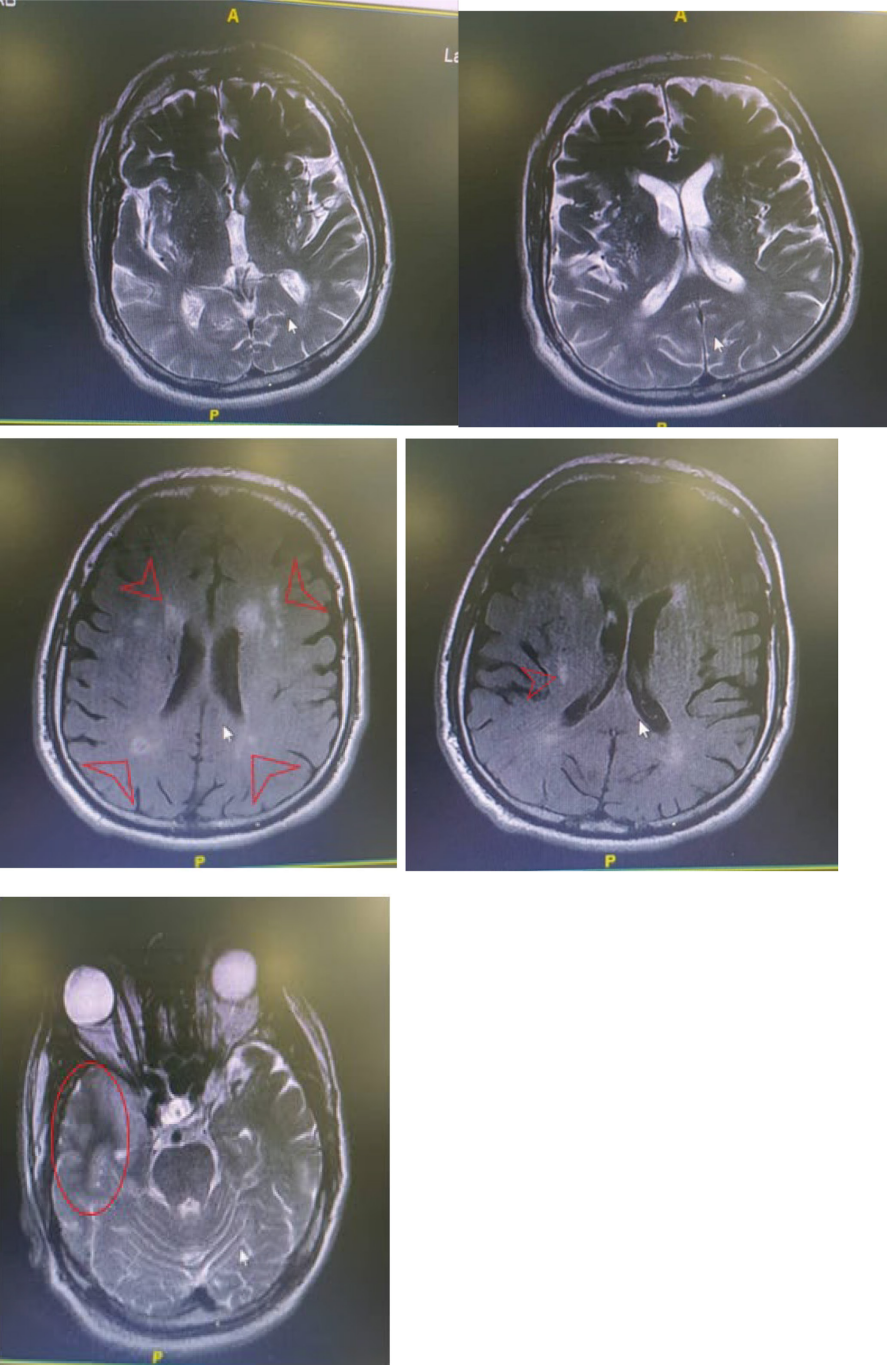
MRI of brain. Several T2, FLAIR hyperintense area is seen at deep white matter of both cerebral hemispheres in favor of deep white matter ischemic change (red arrows). Increased signal intensity with sulci effacement is seen at right temporal lobe involving medial and anterior temporal pole showing no post‐Gad enhancement in favor of encephalitis.

Brain magnetic resonance imaging (MRI) was done (Figure 1). The COVID‐19 PCR test was negative. Other laboratory data showed serum sodium (Na) level, 122 mEq/L (first day), and 135 mEq/L (discharge day), white blood cell (WBC) 10,200 per microliter, hemoglobin 11.7 g/dL, platelet 352,000 per microliter, uric acid 3.7 mg/dL, normal liver function test (LFT), C‐reactive protein (CRP) 1, and lumbar puncture (LP), showed no WBC, antinuclear antibody (ANA), Anti ds DNA, C3, C4, C‐ANCA, and P‐ANCA all of them were normal. In the beginning of hospitalization, there was hyponatremia in the result of laboratory test; therefore, management of hyponatremia started. But, the patient developed seizure in less than 24 h that required intubation in the absence of sodium overcorrection. His hyponatremia could not justify the patient's seizures. LP was normal. After 48 h, the patient became extubated and evaluation for the cause of hyponatremia showed euvolemic hyponatremia with normal thyroid stimulating hormone (TSH: 3.1 μg/dL) and cortisol level. Brain MRI favored encephalitis; however, cerebrospinal fluid (CSF) herpes PCR was negative, so syndrome of inappropriate antidiuretic hormone secretion (SIADH) due to encephalitis post vaccine was the most probable cause of hyponatremia according to International Encephalitis Consortium criteria^10^ (Table 2).

**TABLE 2:** Diagnostic criteria for encephalitis according to the 2013 International Encephalitis Consortium.

Diagnostic criteria	Our patient
Major criterion (required)	
Altered mental status lasting 24 h or more (not attributable to other diseases)	✓
Minor criteria (two for possible, ≥3 for probable or confirmed diagnosis)	
Temperature ≥38°C within 72 h before or after manifestations	✓
Seizures (generalized or partial) not attributable to other diseases	✓
New onset focal neurological deficits	✗
Leukocyte count in cerebral spinal fluid ≥5 mm^3^	✗
Neuroimaging findings	✓
Electroencephalography findings	–

### 2.1 Differential diagnosis

Viral encephalitis, post‐COVID‐19 encephalitis, hypersensitivity reaction to vaccine.

### 2.2 Treatment

The patient received levetiracetam (150 mg Q12h) for seizure. Also received three pulses of methylprednisolone 1 g and 120 g intravenous immunoglobulin (IVIG), which were discontinued after discharged.

### 2.3 Outcome and follow‐up

His condition improved, and he became oriented to time, place, and person. He was discharged from the hospital and at follow‐up, and he was asymptomatic and fully oriented without convulsion.

## 3 DISCUSSION

This report described a 65‐year‐old previously healthy man who developed high‐grade fever, hallucination, and disorientation a day after getting his second dose of COVID‐19 Sinopharm vaccine. Also, the patient developed seizures and laboratory findings showed hyponatremia; however, it could not justify the patient's seizures. Brain MRI favored encephalitis, and CSF PCR for herpes was negative, so SIADH due to encephalitis post‐vaccine was the most probable cause of hyponatremia. The patient received methylprednisolone and IVIG and showed improvement, supporting an immune‐mediated mechanism behind his acute presentation.

Encephalitis is an inflammatory neurological disorder with several etiologies that typically occurs after infections or vaccination.^11^ Recently, acute disseminated encephalomyelitis (ADEM) has also been associated with COVID‐19 vaccination.^12^ However, encephalitis after COVID‐19 vaccination is rare and only few cases have been reported worldwide; our patient case presentation findings were consistent with documented results in published literature (Table 3). Some studies described cases who developed encephalitis after the Moderna vaccine.^7^, ^13^, ^14^ Also, the others reported patients who exhibited encephalitis presentation after receiving the Pfizer vaccine.^15^, ^16^, ^17^ Furthermore, some case reports presented cases with encephalitis who received the AstraZeneca and COVISHIELD, which suggests cytokine storm as its causative mechanism.^11^, ^12^, ^18^, ^19^ It demonstrates that ADEM should be considered in patients developing neurological symptoms after COVID‐19 vaccination, although this is rare.^12^ Also, transient amnesia as a result of autoimmune encephalitis after COVID‐19 vaccination has been reported as a less frequent neurological complication.^20^

**TABLE 3:** Characteristics of the studies.

Study	Case	Presentation	Type of vaccine	Treatment	Outcome
Current study	65 years/man	Fever Hallucination Disorientation	Sinopharm; 24 h after the second dose	Steroid IVIG	Improvement
Baldelli L (18)	77 years/man with Sarcoidosis Polymyalgia rheumatica	Confusion Agitation, Delirium Fever	AstraZeneca; 24 h after the first dose	Steroid	Improvement
Al‐Quliti K (11)	56 years/woman	Gradual discomfort Weakness Myalgia Speech difficultly	AstraZeneca; 10 days after the first dose	Steroid	Improvement
Permezel F (12)	63 years/man with Diabetes Ischemic heart disease	Vertigo Abdominal pain Fatigue	Oxford /AstraZeneca; 12 days after the first dose	Antibiotic & antiviral	Dead
Malhotra H.S (19)	36 years/man	Abnormal sensations in lower limbs and trunk	Oxford/AstraZeneca, COVISHIELDTM; 8 days after the first dose	Steroid	Improvement
Senda J (15)	72 years/woman with Rheumatoid vasculitis	Depressed consciousness	Pfizer; 3 days after the first dose	Steroid Gammaglobulin	Improvement
Assiri S.A (16)	80 years/woman with Hypertension Diabetes, Epilepsy	Seizures, Vertigo Dysphagia Dysarthria	Pfizer; 16 days after the second dose	–	Improvement
Vogrig A (17)	56 years/woman	Unsteady gait Clumsiness of arm Malaise, Chills	Pfizer; 14 days after the first dose	Steroid	Improvement
Al‐Mashdali A.F (7)	32 years/man	Confusion Memory disturbance Hallucination	Moderna; 24 h after the first dose	Steroid	Improvement
Rastogi A (13)	59 years/woman	Unsteady gait Incoordination Lethargy	Moderna; 12 days after the second dose of vaccination but the first dose of Moderna	No treatment	Improvement
Gao JJ (14)	82 years/woman	Fever Headache Behavior change	mRNA‐1273; 5 days after the first dose developed fever and headache, 17 days after the first dose developed behavior change	Steroid	Improvement

Considering presence of prodromal symptoms before receiving COVID‐19 vaccine and then progressing to encephalitis, it should be noted that persistent adverse outcomes were three times higher by taking any dose of COVID‐19 vaccine, post‐COVID‐19 or COVID‐19‐like symptoms, that represented the need for closer scrutiny in these patients.^21^ Although the occurrence of encephalopathy after vaccination may be just a casual temporal association, the cytokine storm could result from an excessive innate immune response against the vaccine in a predisposed patient susceptible to autoimmunity.^18^

A possible mechanism in occurrence of post‐COVID‐19 rapid neurological manifestations such as rapidly progressive dementia can be the role of cross‐reactive antibodies after COVID‐19 vaccine.^22^

Acute transverse myelitis and demyelinating polyneuropathy are another rare neurological complications initiated by mimicry molecular phenomenon after COVID‐19 vaccination.^23^

Although vaccines are considered some of the safest and most effective drugs, but adverse reactions are unavoidable, especially during a pandemic,^16^ hence further research is needed to clarify the pathophysiology of such complications.

## 4 CONCLUSION

Present report described encephalitis after receiving the COVID Sinopharm vaccine. In this patient, considering presence of prodromal symptoms before receiving COVID‐19 vaccine and then progressing to encephalitis, we think that vaccination might have triggered the cascade in the presence of underlying prodrome and should be avoided during the time of prodromal symptoms. Suspicion for rare but serious neurological features should be highlighted for timely detection and prevention of complications along with need of real‐world comparative studies between vaccinated and unvaccinated.

## AUTHOR CONTRIBUTIONS

**Maryam Pakfetrat:** Conceptualization; data curation; investigation; methodology; project administration; supervision; validation; visualization; writing – original draft. **Leila Malekmakan:** Data curation; investigation; methodology; resources; supervision; validation; writing – review and editing. **Bijan Najafi:** Conceptualization; data curation; methodology; project administration; resources; validation; visualization; writing – original draft. **Taraneh Zamani:** Data curation; formal analysis; project administration; supervision; validation; visualization; writing – original draft; writing – review and editing. **Mina Mashayekh:** Visualization; writing – original draft; writing – review and editing.

## FUNDING INFORMATION

None.

## CONFLICT OF INTEREST

The authors declare that they have no conflict of interest. The authors received no financial support for the research, authorship, and/or publication of this article.

## CONSENT

Written informed consent was obtained from the patient to publish this report in accordance with the journal's patient consent policy.

## Data Availability

All data are presented within the manuscript file.
